# Effect of Zinc Supplementation on Growth Outcomes in Children under 5 Years of Age

**DOI:** 10.3390/nu10030377

**Published:** 2018-03-20

**Authors:** Enju Liu, Laura Pimpin, Masha Shulkin, Sarah Kranz, Christopher P. Duggan, Dariush Mozaffarian, Wafaie W. Fawzi

**Affiliations:** 1 Institutional Centers of Clinical and Translational Research, Boston Children’s Hospital, 300 Longwood Ave, Boston, MA 02115, USA; 2 Division of Gastroenterology, Hepatology and Nutrition, Boston Children’s Hospital, 300 Longwood Ave, Boston, MA 02115, USA; christopher.duggan@childrens.harvard.edu; 3 Friedman School of Nutrition Science and Policy, Tufts University, 150 Harrison Ave, Boston, MA 02111, USA; laura.pimpin@gmail.com (L.P.); shulkinm@gmail.com (M.S.); Sarah.Kranz@tufts.edu (S.K.); Dariush.Mozaffarian@tufts.edu (D.M.); 4 Department of Nutrition, Harvard T.H. Chan School of Public Health, Harvard T.H. Chan School of Public Health, 677 Huntington Ave, Boston, MA 02115, USA; 5 Department of Global Health and Population, Harvard T.H. Chan School of Public Health, 677 Huntington Ave, Boston, MA 02115, USA; mina@hsph.harvard.edu; 6 Department of Epidemiology, Harvard T.H. Chan School of Public Health, 677 Huntington Ave, Boston, MA 02115, USA

**Keywords:** zinc supplementation, child growth, randomized controlled trial, meta-analysis, systematic review

## Abstract

(1) Background: The effects of zinc supplementation on child growth, and prior reviews of these studies, have shown mixed results. We aim to systematically review and meta-analyze randomized controlled trials evaluating effects of preventive zinc supplementation for 3 months or longer during pregnancy or in children up to age 5 years on pregnancy outcomes and child growth; (2) Methods: We searched PubMed, EMBASE, Cochrane Library, Web of Science, and trial registries for eligible trials up to October 10, 2017. Inclusion selection and data extractions were performed independently and in duplicate. Study quality was evaluated by the Cochrane Risk of Bias tool. Findings were pooled using random effects meta-analysis, with heterogeneity assessed by *I*^2^ and τ^2^ statistic, stratified analyses, and meta-regression, and publication bias by Egger’s and Begg’s tests; (3) Results: Seventy-eight trials with 34,352 unique participants were identified, including 24 during pregnancy and 54 in infancy/childhood. Maternal zinc supplementation did not significantly increase birth weight (weighted mean difference (WMD) = 0.08 kg, 95%CI: −0.05, 0.22) or decrease the risk of low birth weight (RR = 0.76, 95%CI: 0.52–1.11). Zinc supplementation after birth increased height (WMD = 0.23 cm, 95%CI: 0.09–0.38), weight (WMD = 0.14 kg, 95%CI: 0.07–0.21), and weight-for-age *Z*-score (WMD = 0.04, 95%CI: 0.001–0.087), but not height-for-age *Z*-score (WMD = 0.02, 95%CI: −0.01–0.06) or weight-for-height Z score (WMD = 0.02, 95%CI: −0.03–0.06). Child age at zinc supplementation appeared to modify the effects on height (*P*-interaction = 0.002) and HAZ (*P*-interaction = 0.06), with larger effects of supplementation starting at age ≥2 years (WMD for height = 1.37 cm, 95%CI: 0.50–2.25; WMD for HAZ = 0.12, 95%CI: 0.05–0.19). No significant effects of supplementation were found on the risk of stunting, underweight or wasting; (4) Conclusion: Although the possibility of publication bias and small study effect could not be excluded, the current meta-analysis indicates that zinc supplementation in infants and early childhood, but not pregnancy, increases specific growth outcomes, with evidence for a potentially stronger effect after 2 years of age. These findings inform recommendation and policy development for zinc supplementation to improve growth among young children.

## 1. Introduction

Poor childhood growth, including, underweight, stunting, and wasting, remains a global public health challenge [1]. Worldwide, stunting affects an estimated 165 million children; and wasting, 52 million children. Undernutrition in early life is associated with adverse health outcomes, including higher morbidity and mortality, lower school performance, lower work capacity, and worse economic status as an adult [2,3,4]. Zinc, an essential micronutrient distributed throughout the body, has many critical effects for child growth. Zinc participates in cell division and growth, intestinal electrolyte absorption, neurotransmission, immune response, enzymatic catalysis or stabilization, and functional modification of membrane proteins, gene-regulatory proteins, and hormonal receptors [5,6,7]. Through these pathways, zinc contributes to DNA and RNA synthesis, protein metabolism, and overall growth and development [7]. In both animal and human studies, zinc deficiency can restrict growth [8,9,10]. Globally, nearly 1 in 5 (17%) of the world’s population is at risk of zinc deficiency due to inadequate dietary intake of major sources including lean meat, shellfish, and nuts, with Asia and Africa having the highest prevalence [3,11,12]. Thus, zinc supplementation may be a crucial intervention to improve child growth and reduce underweight and stunting globally. 

While several randomized trials have examined the effect of zinc supplementation on child growth outcomes, the results of these trials, as well as prior reviews of these trials, have been mixed. Some reviews concluded that zinc supplementation had a positive effect on child growth [13,14,15,16,17], while others did not [18,19]. Differences may relate to variability in study settings, time period of supplementation (maternal, infancy/childhood), inclusion or exclusion criteria, dose, duration, or type of zinc, presence of iron supplementation, or types of outcomes assessed. Better understanding of the role of zinc to address child undernutrition in the context of these factors will help to inform nutritional policy development around the world to improve child health and growth. 

To address these uncertainties, we performed a systematic review and meta-analysis of randomized controlled trials in which study participants received preventive zinc supplementation for 3 months or longer to improve child growth, including separate analyses by different time periods (maternal, infancy/childhood) and varied growth outcomes, including at birth and childhood height, weight, corresponding *Z*-scores, and risk of underweight, stunting, and wasting.

## 2. Materials and Methods

We followed the Preferred Reporting Items for Systematic Reviews and Meta-Analyses (PRISMA) guidelines [20] during all stages of implementation, analysis, and reporting of this meta-analyses.

### 2.1. Primary Exposure and Outcomes

The exposure of interest was zinc supplementation during pregnancy, in infants (up to 24 months), or in children (up to 5 years). For endpoints measured at birth, the growth outcomes of interest were birth weight and low birth weight (LBW, defined as birth weight < 2500 g). For outcomes measured in children, the growth outcomes of interest were height, weight, corresponding *Z*-scores including height-for-age (HAZ), weight-for-age (WAZ), and weight-for-height (WHZ), and risk of stunting (HAZ < −2), wasting (WHZ < −2), and underweight (WAZ < −2).

### 2.2. Search Strategy

We conducted literature searches of electronic databases including PubMed (www.ncbi.nlm.nih.go/pubmed), EMBASE (www.ovid.com/embase), Web of Science (www.webofknowledge.com), The Cochrane Library (http://www.cochranelibrary.com/), and the international standard randomized control trial number register (http://www.isrctn.com). Examples of search terms included: (zinc OR zinc supplement OR zinc fortification) AND (stunting OR height or birth weight) AND (pregnant women OR infant OR child) AND (randomized OR clinical trial). Complete search terms and strategies for each database are Supplementary Table S1. All years and languages were searched without restriction through 10 October 2017. These electronic searches were supplemented by hand searching of citation lists and electronic searching of the first 20 “related articles” on PubMed for all final included publications; as well contacts with experts to identify any other recently published studies. The title and abstract of all identified references were screened by one investigator (E.L.); and for any potentially relevant manuscript, the full text was independently assessed in duplicate by two investigators (L.P., E.L.) to determine eligibility, with any differences resolved by consensus.

### 2.3. Study Selection

*Inclusion criteria.* We included all randomized controlled trials that reported on the effect of zinc supplementation in pregnancy, infants (age < 2 years), or children (age ≥ 2 years), including premature infants, low birth weight infants, stunted or malnourished children, on birth or child growth outcomes as described above, including an effect measure and information to compute its standard error. 

*Exclusion criteria.* We excluded studies with other intervention components in which the effect of zinc could not be separated between treatment groups due to other unequal interventions, in which the dose of zinc supplementation intake could not be quantitatively measured, or with duration of supplementation < 3 months. To test the hypothesis that zinc supplementation has a meaningful effect on child growth, there should be a threshold in duration of supplementation for zinc to show a relevant and sustained effect. We excluded studies with very short duration because this would be less relevant to the clinical outcome of interest, i.e., meaningful long-term child growth. Given our interest in the sustained effects on child growth, we did not include any limitation for time of follow up. We excluded observational studies, cross-sectional ecological studies, commentaries, general reviews, or case reports; or trials conducted in populations with major chronic disease (e.g., sickle-cell disease, cystic fibrosis, HIV infection, and severe protein energy malnutrition). When duplicate publications from the same study were identified, we included the publication reporting the largest number of participants for each outcome of interest.

### 2.4. Data Extraction

Data from included studies were independently extracted in duplicate by two investigators (L.P., E.L.) using a standardized electronic form (Microsoft Excel), with any differences resolved by consensus. Information was extracted on the publication (first author, contact information, publication year), study details (name, location, year(s) of enrollment), population (age, socioeconomic status, number of participants in treatment and control arms), baseline nutritional status (e.g., proportion of low birth weight or stunting), zinc intervention (type, daily dose, duration), duration of follow-up, age at outcome assessment, dropout rate, and outcomes including effect measures and associated uncertainty. Missing information was obtained by direct author contact or, if necessary, estimated using a standard approach (see Statistical analysis, below). Study quality was assessed using the Cochrane Collaboration risk-of-bias tool for randomized controlled trials, including potential for selection bias, performance bias, detection bias, attrition bias, and reporting bias through a six-question quality control check list [21]. Each question was answered as low (score = 1), high (score = −1), or unclear (score = 0) risk of bias; and values were summed (potential range: −6 to +6). A score of 5–6 was considered to represent high quality; 3–4, medium quality; and −6 to 2, low quality.

### 2.5. Statistical Analysis

For continuous outcomes (e.g., height, HAZ, weight, WAZ, WHZ), mean difference approach was used to pool trials reporting an outcome in same unit. The primary effect measure was the mean difference in changes from baseline to follow-up in the intervention vs. control group. For studies where mean changes from baseline were not reported, the difference in follow-up measures between treatment groups was used. For binary outcomes (e.g., risk of stunting, wasting), odds ratios or relative risks (RRs) were extracted, or calculated based on numbers of events and sample size across treatment groups. The standard error (SE) for each effect measure was extracted or directly calculated from other reported uncertainty measures (standard deviation, 95% confidence interval, P value). We utilized the values from intent-to-treat analysis as the default. For studies in which subjects with missing outcome at follow-up were excluded from the analysis, we included their available published results. For trials reporting effects by stratum (e.g., by sex or randomized factorial design), we calculated the overall study-specific zinc effect by performing a fixed effects meta-analysis of these strata, for each study. Findings from all trials were pooled using random effects inverse-variance weighted DerSimonian and Laird meta-analyses [22].

Heterogeneity was assessed using the *I*^2^ statistic [23], with thresholds of <30%, 30–60%, and >60% considered to represent low, moderate, and high heterogeneity, respectively. We explored in stratified analyses whether pre-specified factors accounted for significant heterogeneity in effects, including time period of initiating supplementation (maternal, infants, children), duration of supplementation, daily dose of zinc supplementation, world region (African, Asian, America, or Western), publication year (<2000, 2000+), residence (rural, urban, or both), and presence or absence of background iron supplementation to both invention and control groups. Statistical significances of potential differences were assessed using meta-regression. In sensitivity analyses, we excluded trials with low quality scores (≤2). Potential for publication bias was evaluated by visual inspection of funnel plots and by Egger’s [24] and Begg’s [25] tests. If publication bias was suggested, we used Duval and Tweedie’s non-parametric “trim and fill method” to estimate the pooled effects adjusted for any hypothetically missing studies. All analyses were performed with STATA 14 (StataCorp, College Station, TX, USA), with 2-tailed alpha = 0.05. 

## 3. Results

### 3.1. Study Characteristics

Among 1107 identified articles, 78 trials met eligibility criteria (Supplementary Figure S1), totaling 34,352 unique participants including 13,167pregnant mothers (24 trials) and 20,412 infants < 2 years old (47 trials), and 773 children aged 2 years or older (7 trials) who received supplementation (Supplementary Tables S2 and S3). Only one trial included was cluster-randomized, the remainder were individually randomized. 15 trials had a factorial design. For trials with co-interventions (given to both groups), 16 of 24 pregnant women trials had co-interventions, most commonly folic acid and iron combined; and 30 of 54 children trial had co-interventions, most commonly iron combined with multi-micronutrients. These 78 trials were conducted on 6 continents, including 7 studies in the US and Caribbean, 1 in Australia, 4 in Europe, 27 in Asia, 19 in South and Central America, 10 in Africa, and 10 in the Middle East (Supplementary Figure S9). The mean age at randomization for pregnant mothers was 25.0 years; for infants, 8.7 months; and for children, 43.4 months. The mean duration of intervention was 22.9 weeks for trials in pregnant women, 30.9 weeks in infants, and 38.9 weeks in children; with mean zinc doses of 26.8, 7.6, and 8.5 mg/day, respectively. The follow-up period for majority of the 78 trials was the same length as the intervention. In only eight trials in children, an extended follow-up after the intervention was over was carried out. For zinc formulation, 54 (69%) of the 78 trials used sulfate zinc; 9 trials (11%), gluconate zinc; 6 (8%) acetate; 6(8%) unknown; the remaining 3 trials (4%) are citrate, lactate and methionine, respectively. According to Cochrane Collaboration risk-of-bias tool for randomized controlled trials, 52 (66.7%) of the 78 trials had a quality score of 5 or 6, classified as high quality, and 11 (14.1%) trials had a score 2 or below, classified as low quality. (Table 1). All trials utilized zinc supplementation. Trials of zinc fortification were evaluated and excluded due to other unequal interventions between treatment groups that would prevent isolation of the effect of zinc.

### 3.2. Maternal Zinc Supplementation and Birth Growth Outcomes

Among 24 trials during pregnancy, 22 reported on birthweight and 13 on LBW (<2500 g) as main outcomes. Zinc supplementation did not significantly affect birthweight (WMD = 0.08 kg; 95%CI: ‒0.05–0.22) or LBW (RR = 0.76 (95%CI: 0.52–1.11) (Supplementary Figures S2 and S3). While statistical heterogeneity was high (*I*^2^ > 90%), 18 of 22 studies of birthweight found no significant effect; and two trials appeared to be outliers. In post-hoc analyses excluding these trials, the WMD was 0·01 (95%CI: −0.01–0.04); with *I*^2^ = 45%. Similarly, ten of 13 trials of LBW found no significant effect; excluding 1 potential outlier trial, the pooled RR was 0.97 (95%CI: 0.79–1.19); with *I*^2^ = 46.7%.

These findings were not significantly different by duration of supplementation, daily dose of zinc supplementation, world region (African, Asian, America, or Western), publication year (<2000, 2000+), residence (rural, urban, or both), or background iron supplementation (Table 2).

### 3.3. Infant and Child Zinc Supplementation and Growth Outcomes

#### 3.3.1. Height, HAZ and Stunting

**Figure 1:**
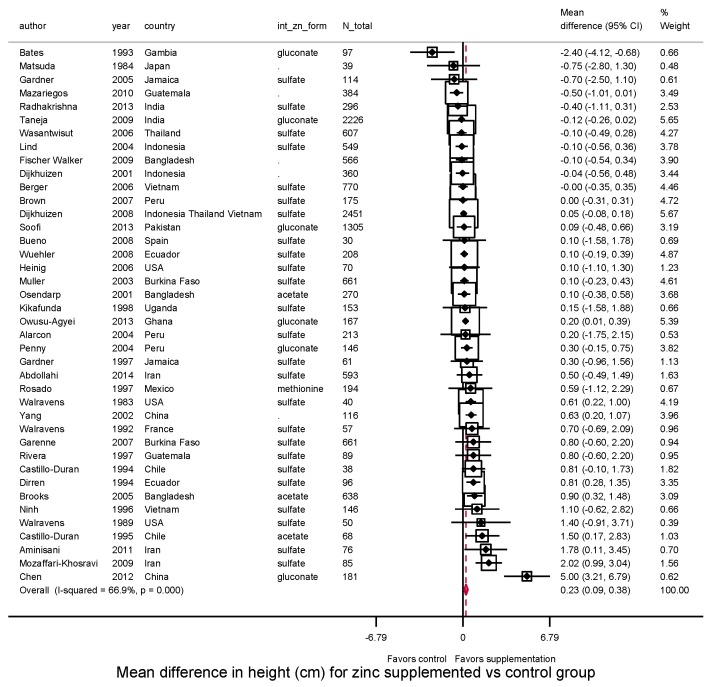
Effect of zinc supplementation among children aged < 5 y old on height in randomized controlled trials.

**Figure 2:**
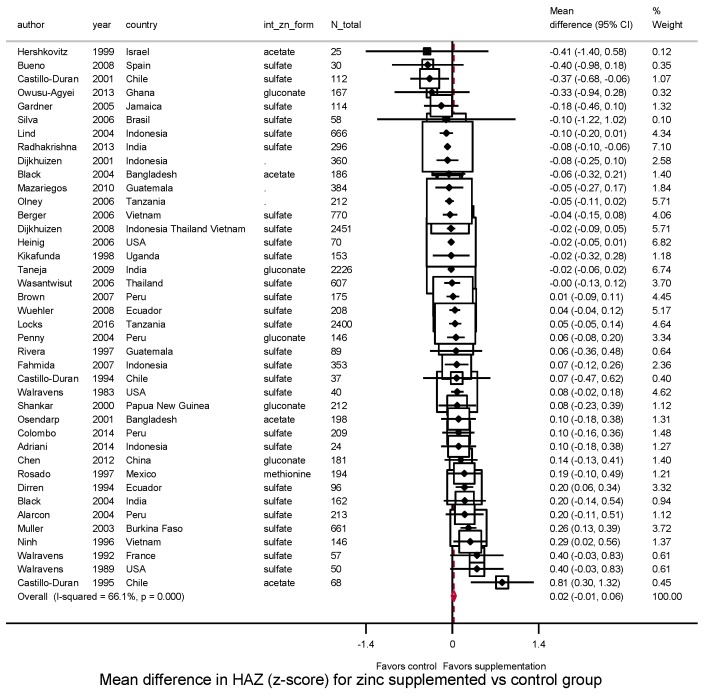
Effect of zinc supplementation among children aged < 5 y old on HAZ in randomized controlled trials.

Among trials conducted after birth, zinc supplementation significantly increased height (*N* = 40 trials, WMD = 0.23 cm, 95%CI: 0.09–0.38; *I*^2^ = 66.9%) (Figure 1). Twenty-eight of 40 studies (70%) had a positive effect size, and 10 were statistically significant. The effect on HAZ was not statistically significant (*N* = 40 trials; WMD = 0.02; 95%CI: −0.01–0.06; *I*^2^ = 65.6%) (Figure 2); about half (22 of 40) reported a positive effect size, and only 4 were statistically significant.

When we explored potential factors that might modify these effects, significant heterogeneity was not identified by world region, duration of supplementation, daily dose of zinc supplementation, residence (rural, urban, or both), socioeconomic status, zinc type, or background iron supplementation (*P*-interaction > 0.05 each) (Table 3). However, zinc had a greater effect on height and HAZ for supplementation in children compared with infants (*P*-interaction = 0.002 and 0.06, respectively). Among children aged ≥2 years, zinc increased height (*N* = 7 trials; WMD = 1.37 cm, 95%CI: 0.50–2.25) and HAZ (*N* = 6 trials; WMD = 0.12, 95%CI: 0.05–0.19). Publication year also appeared significant in meta-regression, with stronger effects reported in trials published before year 2000 than thereafter (*P* = 0.08 for height, *P* = 0.002 for HAZ). In multivariable meta-regression including both child age and publication year in the models, child age was an independent predictor of heterogeneity for height (*P*-interaction < 0.05), while publication year was for HAZ (*P*-interaction < 0.05).

Nine trials evaluated the effect of zinc supplementation on stunting. No statistically significant effect was identified (RR = 1.01, 95%CI: 0.96–1.06; *I*^2^ = 0.0%) (Supplementary Figure S4).

#### 3.3.2. Weight, WAZ, WHZ, Underweight, Wasting

**Figure 3:**
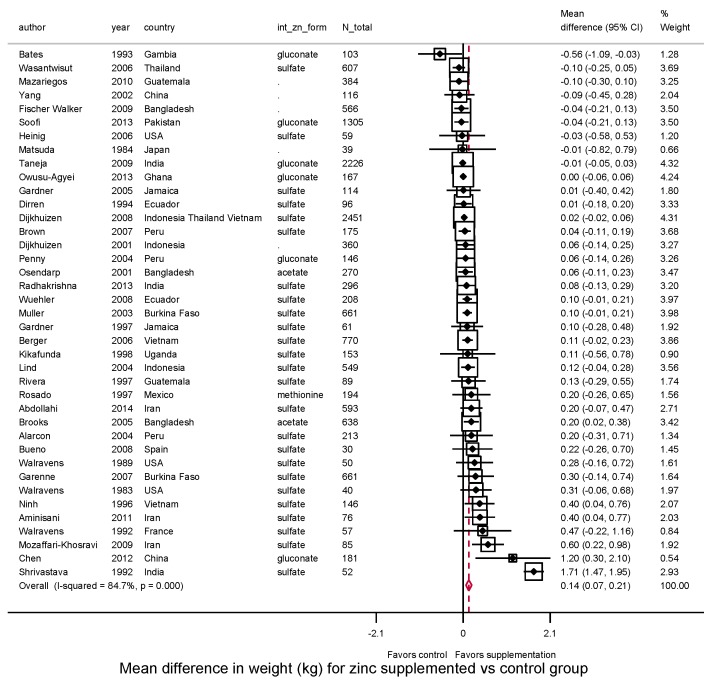
Effect of zinc supplementation among children aged < 5 y old on weight in randomized controlled trials.

**Figure 4:**
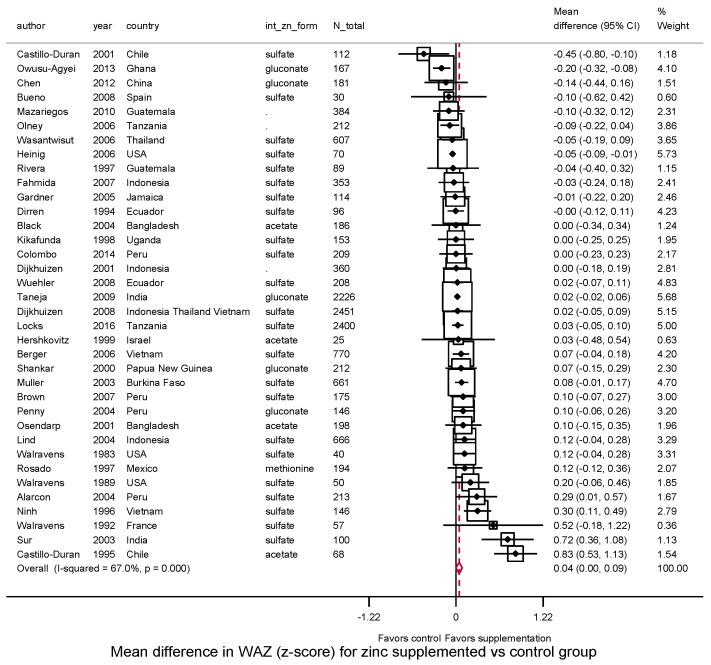
Effect of zinc supplementation among children aged < 5 y old on WAZ in randomized controlled trials.

Zinc supplementation after birth significantly increased weight (*N* = 39, WMD = 0.14 kg, 95%CI: 0.07–0.21, *I*^2^ = 84.7) (Figure 3). Thirty (77%) of 39 trials reported a positive effect size. Supplementation also increased WAZ (*N* = 36, WMD = 0.04, 95%CI: 0.001–0.087, *I*^2^ = 67.2%) with 24 (67%) of 36 trials reporting a positive effect size (Figure 4).

Effects of zinc supplementation were not significantly modified by most underlying participant or study characteristics (Table 4). Similar to HAZ, we found that trials published before year 2000 tended to have a larger effect on WAZ (*P* = 0.03) compared to trials published thereafter.

**Figure 5:**
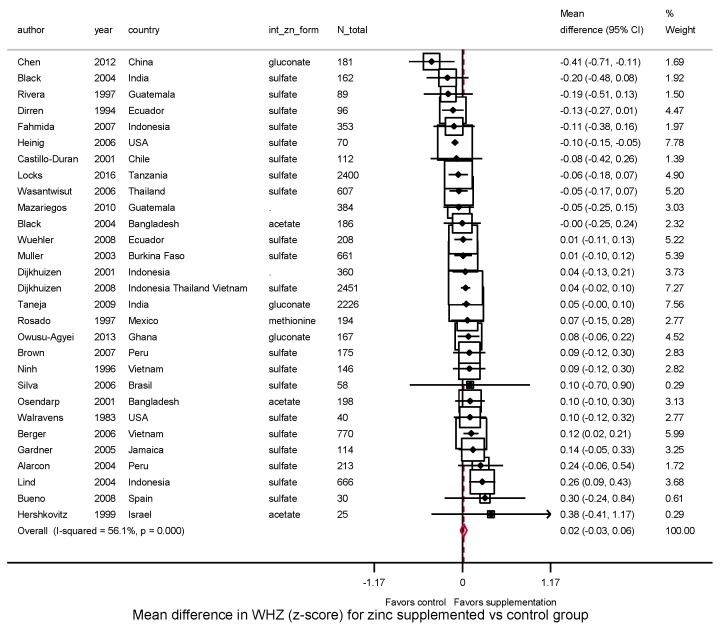
Effect of zinc supplementation among children aged < 5 y old on WHZ in randomized controlled trials.

In 29 trials, zinc supplementation did not significantly affect WHZ (WMD: 0.02, 95%CI: −0.03–0.06, *I*^2^ = 56.1) (Figure 5). Six trials evaluated risk of underweight and seven trials, wasting. Pooling these studies, significant effects were not identified on risk of underweight (RR = 1.03, 95%CI: 0.97–1.09; *I*^2^ = 0.0%) (Supplementary Figure S5) or wasting (RR = 0.88, 95%CI: 0.74–1.05; *I*^2^ = 57.0%) (Supplementary Figure S6).

### 3.4. Influence of Study Quality

In sensitivity analyses, we excluded four maternal trials and six infant/child trials classified as having a low quality score (≤2). Among the remaining trials, zinc supplementation significantly increased height (*N* = 34 trials, WMD = 0.19 cm, 95%CI: 0.05–0.34), weight (*N* = 32, WMD = 0.06 kg, 95%CI: 0.02–0.10), and WAZ (*N* = 33, WMD = 0.06, 95%CI: 0.01–0.10), but not birthweight (*N* = 17, WMD = 0.01, 95%CI: −0.02–0.04), HAZ (*N* = 37, WMD = 0.03, 95%CI: −0.01–0.06), or WHZ (*N* = 26, WMD = 0.02, 95%CI: −0.03, 0.06).

### 3.5. Evaluation of Publication Bias

Visual inspection of funnel plots suggested asymmetry consistent with potential publication bias and small-study effects for height, HAZ, weight, and WAZ (Supplementary Figure S7). Egger’s test identified statistical evidence for potential small-study effects for height (*p* = 0.01), HAZ (*p* < 0.001), weight (*p* = 0.03), and WAZ (*p* = 0.04). In contrast, findings for Begg’s test were not statistically significant for any of these outcomes (*p* ≥ 0.12 each)**.** When we explored the influence of a potential publication bias using the trim-and-fill method, no missing studies were identified for weight or WAZ; while 6 hypothetically missing studies were estimated for height, and 2 for HAZ (Supplementary Figure S8). Addition of these missing studies resulted in a theoretical corrected pooled estimate of 0.14 cm (95%CI: −0.03, 0.31) for height and 0.02 (95%CI: −0.02, 0.05) for HAZ.

## 4. Discussion

In this systematic review and meta-analysis of randomized controlled trials, we found that zinc supplementation in infants and children, but not during pregnancy, improved specific growth outcomes including height, weight, and WAZ. We also identified evidence for potentially stronger effects on height and HAZ by child age, with greater effects when supplements were given to children aged ≥2 years, rather than infants. We did not find evidence for significant effects of zinc supplementation on other growth outcomes including risk of stunting, underweight, or wasting.

Possible small-study effects were seen for height and HAZ, but not other outcomes. This could be due to publication bias or, alternatively, differences in effects of smaller studies from true heterogeneity in certain populations or study designs studying these outcomes. 

During the last trimester of pregnancy, the mother transfers up to 1.5 mg/kg of zinc every day to the fetus, in whom it is mainly stored in fetal liver [26]. Clinical zinc deficiency in preterm neonates has been well documented, characterized by growth impairment and dermatitis [27,28]. The lack of significant effect of zinc supplementation in pregnant women on birth growth outcomes suggests that zinc may have smaller effects in the absence of clinical zinc deficiency.

Our results suggest that benefits of zinc supplementation for height, HAZ and weight might be more effective among children aged ≥ 2 years. The smaller effect in infants could be due to maternal breastfeeding, which provides zinc from the mother [29], or better initial zinc body stores from in utero development, compared to later in life. In the current meta-analysis, most trials did not assess zinc status at enrollment, making it difficult to know if results would vary based on baseline zinc levels. A smaller effect during infancy could also relate to in-field challenges of reliably measuring growth, especially WHZ, during infancy compared with childhood [30].

A smaller effect among infants could also relate to more consistent and rapid growth in the first year of life, compared to later years in which declines in weight or height become more apparent [31]. In undernourished populations, growth often remains rapid early in life, and then begins to falter. This growth pattern might provide some insight as to why zinc supplementation may be more effective among children than infants. First, rapid growth in the first year may lead to negative zinc balance, increasing benefits of supplementation thereafter. Second, most infants have been weaned or are weaning from breastfeeding in their second year of life, making diet a crucial source for zinc intake.

A prior meta-analyses of zinc supplementation in children aged <5 years, published in 2009, identified 43 trials and found no significant effect on height or weight [18]. A second meta-analysis, published in 2011, included 36 trials and found a positive effect only on linear growth, measured by height or HAZ [15]. Other prior meta-analyses [13,14,16] included trials of zinc supplementation throughout childhood, up to age 12 years, and found that zinc supplementation was associated with a small, but significant increase in height and weight; in subanalysis, these benefits persisted in groups aged 1–<5 and 5–<13 years, but not 6–<12 months [16].

Our novel findings build upon and advance these prior results in several ways. In the current analysis, with more trials included, we found that zinc supplementation not only increases height and HAZ, especially among those older than 24 months, but also has a positive effect on weight and WAZ. We included multiple time periods of supplementation, including pregnancy, infancy, and early childhood, providing the most comprehensive evidence to-date on zinc supplementation and early growth. We separately considered several relevant endpoints including birthweight, height and weight gain, and corresponding Z scores. We conducted separate analysis for height vs. HAZ, and weight vs. WAZ, rather than combining these different measures together as has been done in some prior meta-analyses [15]. Heterogeneity by underlying subject and study characteristics was carefully explored, providing hypothesis-generating evidence on potential factors which might modify effects or explain variation. We also identified potential evidence for small study effects, and evaluated its impact using trim and fill methods. Our findings cannot exclude the possibility of publication bias, and our results should be interpreted in this light. However, small-study effects (asymmetrical funnel plots) cannot be equated with publication bias since such a pattern could also result from other factors including true heterogeneity in effect sizes and differences in populations or methodology [21].

The pooled effect sizes of zinc supplementation on birth weight, height and WHZ appeared potentially much larger in the absence of iron supplementation, although effect modification by background iron supplementation was not statistically significant in meta-regression analyses (*P*-interactions ranging from 0.10 to 0.21). Given physiologic evidence for potential competition for absorption from gut between iron and zinc [32,33,34], our findings, while not achieving statistical significance, support concern for potential interaction and suggest that zinc may not be optimally effective when iron is also supplemented. These novel results highlight the need for future studies to consider and assess competition between iron and zinc supplements for child growth. Our findings, based on the available literature, does not suggest that region (Africa, Asia, western or Americas) modifies the effect of zinc supplementation on child growth outcomes; nor did we identify evidence for modification of effects by different doses or durations, within the ranges tested in these trials. 

Potential limitations should be considered. As with all meta-analyses, our findings are based on available studies and their measurements; fewer trials, for examples, reported binary outcomes such as risk of underweight, wasting, or stunting. On the other hand, our comprehensive literature search of multiple databases together with citations of related articles made it unlikely that we missed any major studies and maximized statistical power. We were not able to identify all sources of statistical heterogeneity, and residual differences could be due to unknown factors or chance. We cannot exclude the possibility of differential effect among infants who were born with low birthweight or who were malnourished after birth given limited studies that examined these subgroups of children. We did not formally assess whether long-term studies might have recruited a subset of subjects who were acutely ill (e.g., diarrhea or pneumonia) at enrollment, which might have temporarily reduced initial zinc bioavailability. We did not review the potential side effects of zinc supplementation or biochemical indicators such as serum or plasma zinc concentration, which are relevant questions for future investigations. Also, since most trials did not have data on baseline plasma zinc concentrations, we were not able to examine the influence of baseline zinc status on the effect of zinc supplementation.

In conclusion, our systematic review and meta-analysis of randomized controlled trials indicates that zinc supplementation in children improves specific growth outcomes, with potentially stronger effects of supplementation in children after age 2 years. Our findings support a role of zinc for certain child growth outcomes in infants and children under five years of age. The modest effect size we identified may not justify universal zinc supplementation. However, larger effects may be observable among children with sub-optimal zinc status. Our results also highlight a need for further trials to confirm the potential stronger benefit on child growth after age of two years, especially as most existing trials focused on the first 1000 days of life. Our novel findings inform policy recommendations and program development for zinc supplementation to improve growth among young children.

## 5. Conclusions

In conclusion, our systematic review and meta-analysis of randomized controlled trials indicates that zinc supplementation in children improves specific growth outcomes, with potentially stronger effects of supplementation in children after age 2 years. Our findings support a role of zinc for certain child growth outcomes in infants and children under five years of age. The modest effect size we identified may not justify universal zinc supplementation. However, larger effects may be observable among children with sub-optimal zinc status. Our results also highlight a need for further trials to confirm the potential stronger benefit on child growth after age of two years, especially as most existing trials focused on the first 1000 days of life. Our novel findings inform policy recommendations and program development for zinc supplementation to improve growth among young children.

## Figures and Tables

**Table 1:** Summary of 78 randomized controlled trials included in the meta-analysis of the effect of zinc supplementation during pregnancy, infancy, or childhood on growth outcomes.

	Pregnant Mothers	Infants (<2 Years)	Children (2–5 Years)
Trials ^1^, *n*	24	47	7
Total participants, *n*	13,167	20,412	773
Subject socioeconomic status ^2^	Low, 14; Medium, 8; High, 2;	Low, 37; Medium, 6; High, 3; -, 1	Low, 5; Medium, 1; High, 0; -, 1
Mean age (range)	25.1 (15.9–30.4) years	8.7 (0–23.5) months	43.4 (28.7–55.8) months
Mean gestational age, weeks (range)	16.0 (9.8–24.0)		
Mean supplement duration, weeks (range)	22.9 (16.0–29.0)	30.9 (12.0–78.0)	38.9 (26.0–64.5)
Mean duration to last f/u, weeks (range)	22.9 (16.0–29.0)	34.7 (12.0–87.0)	43.8 (26.0–64.5)
Mean zinc dose, mg/day (range)	26.8 (10.0–50.0)	7.6 (2.0–20.0)	8.5 (0.37–20.0)
Zinc Formulation	Acetate, 1; Citrate, 1, Gluconate, 2; Lactate, 1; Sulfate 19;	Acetate, 5; Gluconate 6; Sulfate 30, unknown 6	Gluconate, 1; Methionine, 1; Sulfate, 5
Growth outcomes	BW, LBW	Weight, Height, WAZ, WHZ, HAZ, stunting, wasting, underweight	Weight, Height, WAZ, WHZ, HAZ, stunting, wasting, underweight
Quality score ^3^, *n* trials	Low, 4; Medium, 3; High, 15	Low, 6; Medium, 10; High, 31	Low, 1; Medium, 0; High, 6

LBW = Low birth weight; WAZ = Weight-for-age; WHZ = Weight-for-Height; HAZ = Height-for-age; BW = Birthweight; - indicates information is unavailable. ^1^ All studies were randomized controlled trials. Most were also placebo-controlled, except for 1 open-label trial in pregnant mothers, 1 in infants, and 1 in children. ^2^ If not reported in the text, socioeconomic status was estimated based on study characteristics, determined by two reviewers independently and in duplicate. “–“stands for missing. ^3^ The Cochrane Collaboration’s tool for assessing risk of bias was used to score studies as having a low (−6 to 2), medium (3–4), or high score (5–6) using a 12-question form.

**Table 2:** Main and subgroup analyses of the effects of zinc supplementation during pregnancy on birth weight.

	Birth Weight(kg)				
	*n*	Mean Difference (95%CI)	*I*^2^	τ^2^	*P*-interaction ^1^
Overall	22	0.08	98.9%	0.10	
		(−0.05, 0.22)			
World region					
Africa	3	0.01	0.0%	0.00	0.65
		(−0.02, 0.04)			
Asia	11	0.14	99.3%	0.14	
		(−0.08, 0.37)			
Western	4	0.03	0.0%	0.00	
		(−0.00, 0.07)			
Americas	4	0.02	27.1%	0.00	
		(−0.03, 0.07)			
Rural/urban residence					
Rural	3	0.01	79.7%	0.00	0.60
		(−0.07, 0.10)			
Urban	14	0.12	99.1%	0.13	
		(−0.07, 0.31)			
Both	1	−0.04	NA	0.00	
		(−0.15, 0.08)			
Unknown	4	0.02	16.8%	0.00	
		(−0.01, 0.06)			
Socioeconomic status					
Lower	14	0.06	99.2%	0.13	0.74
		(−0.13, 0.25)			
Medium	6	0.14	97.1%	0.06	
		(−0.07, 0.34)			
Higher	2	0.04	0.0%	0.00	
		(0.01, 0.08)			
Intervention duration					
<26 weeks	13	0.09	99.2%	0.13	0.78
		(−0.11, 0.29)			
≥26 weeks	9	0.07	95.8%	0.04	
		(−0.06, 0.20)			
Intervention dose					
<28.3 mg/day	13	0.07	93.3%	0.02	0.67
		(−0.02, 0.15)			
≥28.3 mg/day	9	0.11	99.5%	0.16	
		(−0.16, 0.37)			
Zinc formulation					
Acetate	1	−0.04	NA	0.00	0.78
		(−0.12, 0.03)			
Citrate	1	0.17	NA	0.00	
		(−0.15, 0.49)			
Gluconate	2	0.2	98.6%	0.09	
		(−0.22, 0.62)			
Lactate	1	0.18	NA	0.00	
		(0.04, 0.33)			
Sulfate	17	0.07	99.1%	0.11	
		(−0.09, 0.23)			
		
Background iron supplementation					
No	9	0.16	99.4%	0.13	0.10
		(−0.08, 0.40)			
Yes	13	0.01	49.6%	0.00	
		(−0.02, 0.04)			
Publication year					
Before 2000	6	0.14	99.5%	0.18	0.34
		(−0.21, 0.49)			
2000 and after	16	0.06	92.8%	0.02	
		(−0.01, 0.13)			
Quality score ^2^					
≤2	4	0.32	99.3%	0.11	0.003
		(−0.01, 0.65)			
3~4	2	−0.03	0.0%	0.00	
		(−0.09, 0.03)			
≥5	16	0.02	40.6%	0.00	
		(−0.01, 0.04)			

^1^ *p* value for heterogeneity between subgroups based on meta-regression analysis; ^2^ Cumulative score (out of −6 to +6) on Cochrane Risk of Bias tool.

**Table 3:** Main and subgroup analyses of zinc supplementation during infancy/childhood on height and HAZ.

	Height (cm)					HAZ				
	*n*	MD (95%CI)	*I*^2^	τ^2^	*P*-interaction ^1^	*n*	MD (95%CI)	*I*^2^	τ^2^	*P*-interaction ^1^
Overall	40	0.23	66.9%	0.10		40	0.02	66.1%	0.00	
		(0.09–0.38)					(−0.01, 0.06)			
Child age at intervention										
0–<2 years	33	0.10	44.7%	0.03	0.002	34	0.01	64.6%	0.00	0.06
		(−0.02 0.22)					(−0.03, 0.04)			
2–5 years	7	1.37	82.0%	0.82		6	0.12	0.0%	0.00	
		(0.50, 2.25)					(0.05, 0.19)			
World region										
Africa	5	0.07	58.4%	0.09	0.86	5	0.05	78.9%	0.02	0.63
		(−0.35, 0.49)					(−0.09, 0.19)			
Asia	17	0.26	78.4%	0.13		15	−0.02	49.2%	0.00	
		(0.03, 0.48)					(−0.06, 0.02)			
Western	6	0.53	0.0%	0.00		6	0.05	61.0%	0.10	
		(0.19–0.88)					(−0.07, 0.18)			
Americas	12	0.25	48.8%	0.09		14	0.06	49.6%	0.10	
		(−0.03, 0.53)					(−0.03, 0.14)			
Rural/urban residence										
Rural	15	0.07	51.0%	0.06	0.20	15	0.05	58.4%	0.01	0.79
		(−0.13, 0.27)					(−0.03, 0.13)			
Urban	21	0.55	77.2%	0.27		20	0.02	69.9%	0.00	
		(0.24, 0.86)					(−0.03, 0.07)			
Both	2	0.10	0.0%	0.00		2	−0.01	60.5%	0.00	
		(−0.16, 0.35)					(−0.09, 0.08)			
Unknown	2	0.05	100.0%	1.00		3	−0.03	0.0%	0.00	
		(−0.09, 0.18)					(−0.10, 0.03)			
Socioeconomic status										
Lower	31	0.18	60.6%	0.07	0.43	32	0.03	69.1%	0.01	0.99
		(0.03, 0.32)					(−0.02, 0.07)			
Medium	5	0.88	89.2%	0.46		4	0.03	0.0%	0.00	
		(0.18–1.59)					(−0.03, 0.07)			
Higher	3	0.14	0.0%	0.00		2	0.13	72.3%	0.06	
		(−0.81, 1.08)					(−0.26, 0.53)			
Unknown	1	0.10		0.00		2	0.13	72.3%	0.07	
		(−1.58, 1.78)					(−0.26, 0.53)			
Intervention Duration										
<26 weeks	11	0.11	36.6%	0.03	0.79	9	0.05	39.4%	0.01	0.78
		(−0.10, 0.31)					(−0.06, 0.16)			
≥26 weeks	29	0.27	71.6%	0.15		31	0.02	69.8%	0.01	
		(0.08, 0.48)					(−0.02, 0.06)			
Intervention dose										
<8.4mg/day	18	0.37	74.5%	0.28	0.67	17	−0.01	67.2%	0.00	0.23
		(0.05, 0.70)					(−0.05, 0.04)			
≥8.4 mg/day	22	0.19	58.2%	0.05		23	0.05	53.4%	0.01	
		(0.03, 0.35)					(−0.00, 0.10)			
Zinc formulation										
Acetate	3	0.68	69.9%	0.28	0.86	4	−0.05	0.0%	0.09	0.58
		(−0.06, 1.42)					(−0.11, 0.11)			
		(−0.54, 0.34)					.			
Gluconate	6	0.23	89.2%	0.22		5	−0.01	69.4%	0.00	
		(−0.24, 0.70)					(−0.05, 0.02)			
Methionine	1	0.59	NA	NA		1	0.19	.	0.00	
		(−1.12, 2.29)					(−0.10, 0.49)			
Sulfate	25	0.23	46.0%	0.05		27	0.03	72.3%	0.06	
		(0.06, 0.40)					(−0.01, 0.08)			
Unknown	4	−0.01	75.2%	0.27		3	−0.05	0.0%	0.00	
		(−0.64, 0.61)					(−0.11, 0.01)			
Background iron supplementation										
No	29	0.40	74.9%	0.19	0.11	27	0.05	75.1%	0.01	0.21
		(0.17, 0.63)					(−0.00, 0.10)			
Yes	11	0.02	0.0%	0.00		13	−0.03	0.0%	0.00	
		(−0.08, 0.12)					(−0.06, 0.01)			
Publication year										
Before 2000	13	0.58	30.5%	0.12	0.08	11	0.18	34.6%	0.00	0.002
		(0.20, 0.95)					(0.08, 2.29)			
2000 and after	27	0.15	68.6%	0.08		29	−0.01	60.3%		
		(0.001, 0.30)					(−0.04, 0.03)			
Quality score ^2^										
≤2	6	0.80	83.4%	1.45	0.23	3	−0.07	44.6%	0.02	0.33
		(−0.33, 1.92)					(−0.33, 0.20)			
3~4	7	0.25	60.6%	0.04		9	−0.02	27.7%	0.00	
		(0.03, 0.48)					(−0.07, 0.04)		0.01	
≥5	27	0.18	60.8%	0.11		28	0.04	72.7%	0.00	
		(−0.01, 0.37)					(0.00, 0.09)			

^1^ *p* value for heterogeneity between subgroups based on meta-regression analysis; ^2^ Cumulative score (out of −6 to +6) on Cochrane Risk of Bias tool.

**Table 4:** Main and subgroup analyses of zinc supplementation during infancy/childhood on weight, weight-for-age and weight-for-height *Z* scores.

	Weight (kg)					WAZ					WHZ				
	*n*	MD (95%CI)	*I*^2^	τ^2^	*P*-interaction ^1^	*n*	MD (95%CI)	*I*^2^	τ^2^	*P*-interaction ^1^	*n*	MD (95%CI)	*I*^2^	τ^2^	*P*-interaction ^1^
Overall	39	0.14	84.7%	0.03		36	0.04	67.0%	0.00		29	0.02	56.1%	0.01	
		(0.07, 0.21)					(0.001, 0.087)					(−0.03, 0.06)			
Child age at intervention															
0–<2 years	33	0.12	86.1%	0.03	0.26	31	0.05	70.7%	0.01	0.80	25	0.03	54.1%	0.01	0.17
		(0.05, 0.20)					(−0.00, 0.09)					(−0.02, 0.07)			
2–5 years	6	0.31	61.5%	0.06		5	0.03	0.0%	0.00		4	−0.08	68.2%	0.02	
		(0.03, 0.59)					(−0.05, 0.11)					(−0.26, 0.11)			
World region															
Africa	5	0.03	54.9%	0.01	0.50	5	−0.03	74.3%	0.01	0.66	3	0.01	0.0%	0.00	0.87
		(−0.09, 0.16)					(−0.14, 0.07)					(−0.07, 0.08)			
Asia	18	0.21	92.6%	0.05		13	0.06	55.9%	0.01		12	0.03	54.5%	0.01	
		(0.09, 0.32)					(−0.00, 0.12)					(−0.03, 0.09)			
Western	6	0.23	0.0%	0.00		6	0.06	50.1%	0.01		4	0.03	53.7%	0.02	
		(0.03, 0.44)					(−0.08, 0.19)					(−0.16, 0.22)			
Americas	10	0.05	0.0%	0.00		12	0.07	73.7%	0.02		10	0.01	14.5%	0.00	
		(−0.01, 0.12)					(−0.04, 0.18)					(−0.07, 0.08)			
Rural/urban residence															
Rural	15	0.04	40.2%	0.00	0.16	14	0.02	55.5%	0.01	0.44	13	0.03	42.1%	0.01	0.80
		(−0.02, 0.10)					(−0.04, 0.09)					(−0.04, 0.09)			
Urban	20	0.28	91.4%	0.13		17	0.09	78.4%	0.01		12	−0.01	67.1%	0.01	
		(0.1 0–0.46)					(0.02, 0.17)					(−0.09, 0.07)			
Both	2	0.05	46.0%	0.01		2	−0.02	45.9%	0.00		1	0.01	NA	0.00	
		(−0.09, 0.18)					(−0.13, 0.08)					(−0.11, 0.13)			
Unknown	2	0.02	0.0%	0.00		3	0.02	0.0%	0.00		3	0.05	0.0%	0.00	
		(−0.02, 0.06)					(−0.05, 0.08)					(−0.01, 0.11)			
Socioeconomic status															
Lower	30	0.14	87.6%	0.03	0.94	29	0.05	68.7%	0.01	0.91	23	0.03	26.5%	0.00	0.13
		(0.06, 0.22)					(0.00, 0.10)					(−0.00, 0.07)			
Medium	5	0.13	60.0%	0.02		4	0.02	0.0%	0.00		4	−0.04	65.4%	0.03	
		(−0.04, 0.31)					(−0.05, 0.10)					(−0.26, 0.18)			
Higher	3	0.13	0.0%	0.00		2	0.04	70.9%	0.02		1	−0.1	NA	0.00	
		(−0.19, 0.45)					(−0.20, 0.28)					(−0.15, −0.05)			
Unknown	1	0.22	.	0.00		1	−0.10	67.0%	0.01		1	−0.1	NA	0.00	
		(−0.26, 0.71)					(−0.62, 0.42)					(−0.15, −0.05)			
Intervention Duration															
<26 weeks	12	0.30	94.7%	0.09	0.08	8	0.07	74.8%	0.02	0.78	8	0.06	0.0%	0.00	0.10
		(0.10, 0.50)					(−0.06, 0.20)					(0.02, 0.11)			
≥26 weeks	27	0.06	36.9%	0.00		28	0.04	65.4%	0.01		21	0	61.6%	0.01	
		(0.01, 0.11)					(−0.01, 0.09)					(−0.06, 0.05)			
Intervention dose															
<8.4 mg/day	18	0.25	91.3%	0.15	0.15	15	0.05	78.5%	0.02	0.97	12	−0.04	38.3%	0.00	0.03
		(0.05, 0.45)					(−0.04, 0.14)					(−0.10, 0.03)			
≥8.4 mg/day	21	0.04	35.2%	0.00		21	0.04	43.6%	0.00		17	0.05	32.6%	0.00	
		(0.00, 0.08)					(−0.00, 0.08)					(0.01, 0.09)			
Zinc formulation															
Acetate	2	0.13	17.7%	0.00	0.45	4	0.25	83.9%	0.15	0.19	3	0.07	0.0%	0.00	0.96
		(−0.01, 0.27)					(−0.16, 0.67)					(−0.08, 0.22)			
		(−0.21, 0.13)													
Gluconate	6	−0.01	57.6%	0.00		5	−0.03	72.4%	0.01		3	−0.03	77.8%	0.02	
		(−0.08, 0.07)					(−0.14, 0.09)					(−0.20, 0.15)			
Methionine	1	0.20	NA	0.00		1	0.12	NA	0.00		1	0.07	NA	0.00	
		(−0.26, 0.65)					(−0.12, 0.36)					(−0.15, 0.28)			
Sulfate	25	0.23	88.7%	0.01		23	0.05	61.8%	0.01		20	0.02	61.0%	0.01	
		(0.11, 0.34)					(0.00, 0.10)					(−0.04, 0.07)			
Unknown	5	−0.10	0.0%	0.00		3	−0.07	0.0%	0.00		2	0	0.0%	0.00	
		(−0.30, 0.10)					(−0.16, 0.03)					(−0.13, 0.13)			
Background iron supplementation															
No	29	0.18	88.2%	0.05	0.33	24	0.06	73.8%	0.01	0.51	18	−0.01	56.4%	0.01	0.11
		(0.08, 0.29)					(0.00, 0.11)					(−0.07, 0.04)			
Yes	10	0.03	1.3%	0.00		12	0.02	38.5%	0.00		11	0.06	34.3%	0.00	
		(−0.00, 0.07)					(−0.04, 0.08)					(−0.00, 0.12)			
Publication year															
Before 2000	12	0.28	92.7%	0.93	0.16	10	0.19	73.0%	0.04	0.03	6	0	28.9%	0.01	0.72
		(−0.14, 0.69)					(0.04, 0.33)					(−0.12, 0.11)			
2000 and after	27	0.05	41.4%	0.41		26	0.01	57.8%	0.00		23	0.02	61.2%	0.01	
		(0.01, 0.09)					(−0.03, 0.05)					(−0.03, 0.07)			
Quality score ^2^															
≤2	7	0.48	96.9%	0.58	0.04	3	−0.14	16.8%	0.00	0.07	3	−0.03	79.9%	0.04	1.00
		(−0.12, 1.07)					(−0.26, −0.03)					(−0.29, 0.23)			
3~4	6	0.04	0.0%	0.00		6	−0.01	49.7%	0.00		7	0.03	0.0%	0.00	
		(−0.00, 0.07)					(−0.10, 0.07)					(−0.02, 0.08)			
≥5	25	0.06	42.8%	0.01		27	0.08	68.5%	0.01		19	0.02	62.0%	0.01	
		(0.01, 0.12)					(0.03, 0.13)					(−0.04, 0.07)			

^1^ *p* value for heterogeneity between subgroups based on meta-regression analysis; ^2^ Cumulative score (out of −6 to +6) on Cochrane Risk of Bias tool.

## Supplementary Materials

The following are available online at http://www.mdpi.com/2072-6643/10/3/377/s1, Figure S1: PRISMA Flowchart of study selection and inclusion, Figure S2: Effect of zinc supplementation during pregnancy on birth weight in randomized controlled trials, Figure S3: Effect of zinc supplementation during pregnancy on low birth weight in randomized controlled trials, Figure S4: Effect of zinc supplementation among children < 5 years old on risk of stunting in randomized controlled trials, Figure S5: Effect of zinc supplementation among children < 5 years old on risk of underweight in randomized controlled trials, Figure S6: Effect of zinc supplementation among children < 5 years old on risk of wasting in randomized controlled trials, Figure S7: Funnel plots for height (A), HAZ (B), weight (C),WAZ (D), and WHZ (E), Figure S8: Filled funnel plots for (a) height and (b) HAZ using Duval and Tweedie’s non-parametric trim-and-fill method, Figure S9: A global map with all study locations (*n* = 78) included in the meta-analysis, Table S1: Search terms for the meta-analysis in Pubmed, Web of science, Embase, and Cochrane library, Table S2: Characteristics of maternal trials; Table S3: Characteristics of infant and child trials.
