# Supplementary material for: Effect of Zinc Supplementation on Growth Outcomes in Children under 5 Years of Age
Source: Nutrients. 2018 Mar 20;10(3):377. doi: 10.3390/nu10030377 (PMC5872795; doi:10.3390/nu10030377)
Supplement: Supplementary file 1 [file nutrients-10-00377-s001.pdf]

Supplementary Table 1. Search terms for the meta-analysis in Pubmed, web of science, Embase, and Cochrane library.

Database	Search terms
Pubmed	("Zinc"[Mesh] OR "zinc"[tiab] OR "zinc supplement*"[tiab] OR "zinc fortified"[tiab] OR "zinc fortification*"[tiab]) AND ("stunting"[tiab] OR "body height"[mesh] OR "height"[tiab] OR "growth"[mesh] OR "Child Development"[Mesh] OR "Pregnancy Outcome"[Mesh] OR "Birth Weight"[tiab] OR "Premature Birth"[Mesh] OR "Infant, Small for Gestational Age"[Mesh] OR "Small for Gestational Age"[tiab]) AND ("children"[tiab] OR "child"[tiab] OR "infants"[tiab] OR "infant"[tiab] OR "pregnant"[tiab] OR "pregnancy"[tiab] OR "maternal"[tiab] OR "prenatal"[tiab]) AND (Clinical Trial[ptyp] OR trial[tiab] OR randomized[tiab])
Web of science	ts=zinc AND TS=("infant development" OR "stunting" OR "height" OR "child development" OR "growth" OR "birth outcome*" OR "Pregnancy outcome*" OR "birth weight") and TI=("trial*" OR "randomization" OR "randomized" OR "intervention*")
Embase	'zinc'/exp OR 'zinc' AND ('growth disorder'/exp OR 'growth disorder' OR 'growth'/exp OR 'growth' OR 'stunting'/exp OR 'stunting' OR 'height'/exp OR 'height' OR 'birth weight'/exp OR 'birth weight' OR 'birth outcome'/exp OR 'birth outcome' OR 'pregnancy outcome'/exp OR 'pregnancy outcome') AND ('child'/exp OR 'child' OR 'pregnant women'/exp OR 'pregnant women') AND [embase]/lim AND ('trial':ti OR 'trials':ti OR 'randomized':ti OR 'randomization':ti OR 'intervention':ti OR 'interventions':ti)
Cochrane library	'TI Zinc'

Supplementary Table 2. Characteristics of maternal trials.

First author	Year	Country	Sample size	Intervention duration, weeks	Dose, mg/day	Mean maternal age,years	Mean initial gestational age, weeks	Background iron	Study quality score*
Hunt	1985	USA	107	20	20	15.9	16.9	No	4
Mahomed	1989	UK	491	17	20	26.3	20	No	6
Simmer	1991	Australia	52	26	22.5	26	.	No	5
Garg	1993	India	162	24	45		24	No	-2
Goldenberg	1995	USA	580	20	25	23.4	19.2	No	4
Jonson	1996	Denmark	1206	17	44	28.3	20	No	6
Caulfield	1999	Peru	957	23	15	24.6	16.1	Yes	4
Osendarp	2000	Bangladesh	410	28	30	23.1	14	No	6
Castillo-Duran	2001	Chile	507	22	20	16.3	16.9	Yes	5
An	2001	China	97	16	10	24.7		Yes	2
Christian	2003	Nepal	1307	27	30	24	11.4	Yes	6
Qiu	2004	China	670	28	10.5	28	12	No	2
Merialdi	2004	Peru	222	26	25	28.4	13.4	Yes	5
Merialdi	2004	Peru	195	26	25	23	13	Yes	6
Dijkhuizen	2004	Indonesia	136	20	30	25.1	16.3	Yes	5
Hafeez	2005	Pakistan	242	26	20	25.7	12.95	Yes	5
Aminisani	2009	Iran	175	20.5	50	23.9	13	Yes	5
Saaka	2009	Ghana	543	23	20	26.9	13	Yes	6
Danesh	2010	Iran	84	27	50	28.7	14	No	6
Prawirohartono	2013	Indonesia	1956	20	20	28	22	No	6
Nossier	2015	Egypt	392	24	30	26.54	15.6	Yes	6
Sorouri	2015	Iran	528	24	15	28	16	Yes	1
Shahnazi	2017	Iran	92	16	40	30.4	23	No	6
Darling	2017	Tanzania	2056	29	25	22.5	9.8	Yes	6

* Study quality was assessed using the Cochrane Collaboration risk-of-bias tool for randomized controlled trials, including potential for selection bias, performance bias, detection bias, attrition bias, and reporting bias through a 6-question quality control check list. Each question was answered as low (score=1), high (score=-1), or unclear (score=0) risk of bias; and values were summed (potential range: -6 to +6).

Supplementary Table 3. Characteristics of infant and child trials.

First author	Year	Country	Sample size	Intervention duration, weeks	Dose, mg/day	Mean child age, months	Background iron	study quality score*
Walravens	1983	USA	40	52	10.0	40	No	4
Matsuda	1984	Japan	39	20	2.2	1	No	1
Walravens	1989	USA	50	26	5.7	15.2	No	5
alravens	1992	France	57	13	5.0	5.4	No	5
Shrivastava	1992	India	52	13	5.6	16	No	2
Bates	1993	Gambia	103	65	20.0	17.7	No	6
Castillo-Duran	1994	Chile	38	52	10.0	8.7	No	4
Dirren	1994	Ecuador	96	64.5	8.6	33	No	5
Castillo-Duran	1995	Chile	68	26	3.0	0.1	No	5
Ninh	1996	Vietnam	146	20	10.0	17.6	No	5
Rosado	1997	Mexico	194	52	20.0	28.7	Yes	5
Rivera	1997	Guatemala	89	29.9	10.0	7.6	No	5
Gardner	1997	Jamaica	61	12	5.0	14.1	No	5
Kikafunda	1998	Uganda	153	26	8.6	55.8	No	5
Hershkovitz	1999	Israel	25	12	11.2	6.35	No	5
Shankar	2000	Papua New Guinea	212	46	8.6	20	No	6
Osendarp	2001	Bangladesh	270	20	5.0	0.9	No	5
Dijkhuizen	2001	Indonesia	360	24	7.1	4.2	Yes	6
Castillo-Duran	2001	Chile	112	52	5.0	0.6	No	4
Yang	2002	China	116	52	2.5	3.9	No	3
Muller	2003	Burkina Faso	661	26	10.7	18.2	No	6
Sur	2003	India	100	52	3.6	0.5	No	6
Penny	2004	Peru	146	26	10.0	18.9	No	6
Black	2004	Bangladesh	186	26	2.9	6.5	Yes	5
Alarcon	2004	Peru	213	18	7.0	17.5	Yes	5
Black	2004	India	162	32	4.3	1	Yes	4
Lind	2004	Indonesia	666	26	10.0	6.2	Yes	6
Gardner	2005	Jamaica	114	26	10.0	18.7	No	2
Brooks	2005	Bangladesh	638	43	10.0	5.3	No	5
Berger	2006	Vietnam	770	26	10.0	5.9	Yes	6
Heinig	2006	USA	70	26	5.0	4	No	6
Silva	2006	Brasil	58	17	10.0	23.5	Yes	4
Olney	2006	Tanzania	212	26	7.5	8.6	Yes	3
Wasantwisut	2006	Thailand	607	26	10.0	4.4	Yes	6
Brown	2007	Peru	175	26	3.0	7.5	Yes	4
Garenne	2007	Burkina Faso	661	26	10.7	18	No	2
Fahmida	2007	Indonesia	353	26	10.0	5.1	No	6
Dijkhuizen	2008	Indonesia Thailand Vietnam	2451	26	8.6	5.2	Yes	4
Wuehler	2008	Ecuador	208	26	7.0	20.9	No	4
Bueno	2008	Spain	30	26	3.0	0	No	4
Fischer Walker	2009	Bangladesh	566	26	2.9	6.3	Yes	5
Mozaffari-Khosravi	2009	Iran	85	26	5.0	39.3	No	5
Taneja	2009	India	2226	24	18.0	15.3	No	5
Taneja	2009	India	1911	50	7.5	0.51	Yes	6
Mazariegos	2010	Guatemala	384	26	5.0	6	No	5
Aminisani	2011	Iran	76	20	5.0	1	No	6
Chen	2012	China	181	26	7.1	53	No	2
Radhakrishna	2013	India	296	52	5.0	4	No	5
Owusu-Agyei	2013	Ghana	167	24	10.0	14.3	No	1

Soofi	2013	Pakistan	1305	52	10.0	6	Yes	6
Colombo	2014	Peru	209	51.6	10.0	6	Yes	6
Adriani	2014	Indonesia	24	26	0.4	54	No	6
Abdollahi	2014	Iran	593	13	5.0	14.5	Yes	-2
Locks	2016	Tanzania	2400	78	8.3	1.37	No	6

* Study quality was assessed using the Cochrane Collaboration risk-of-bias tool for randomized controlled trials, including potential for selection bias, performance bias, detection bias, attrition bias, and reporting bias through a 6-question quality control check list. Each question was answered as low (score=1), high (score=-1), or unclear (score=0) risk of bias; and values were summed (potential range: -6 to +6).

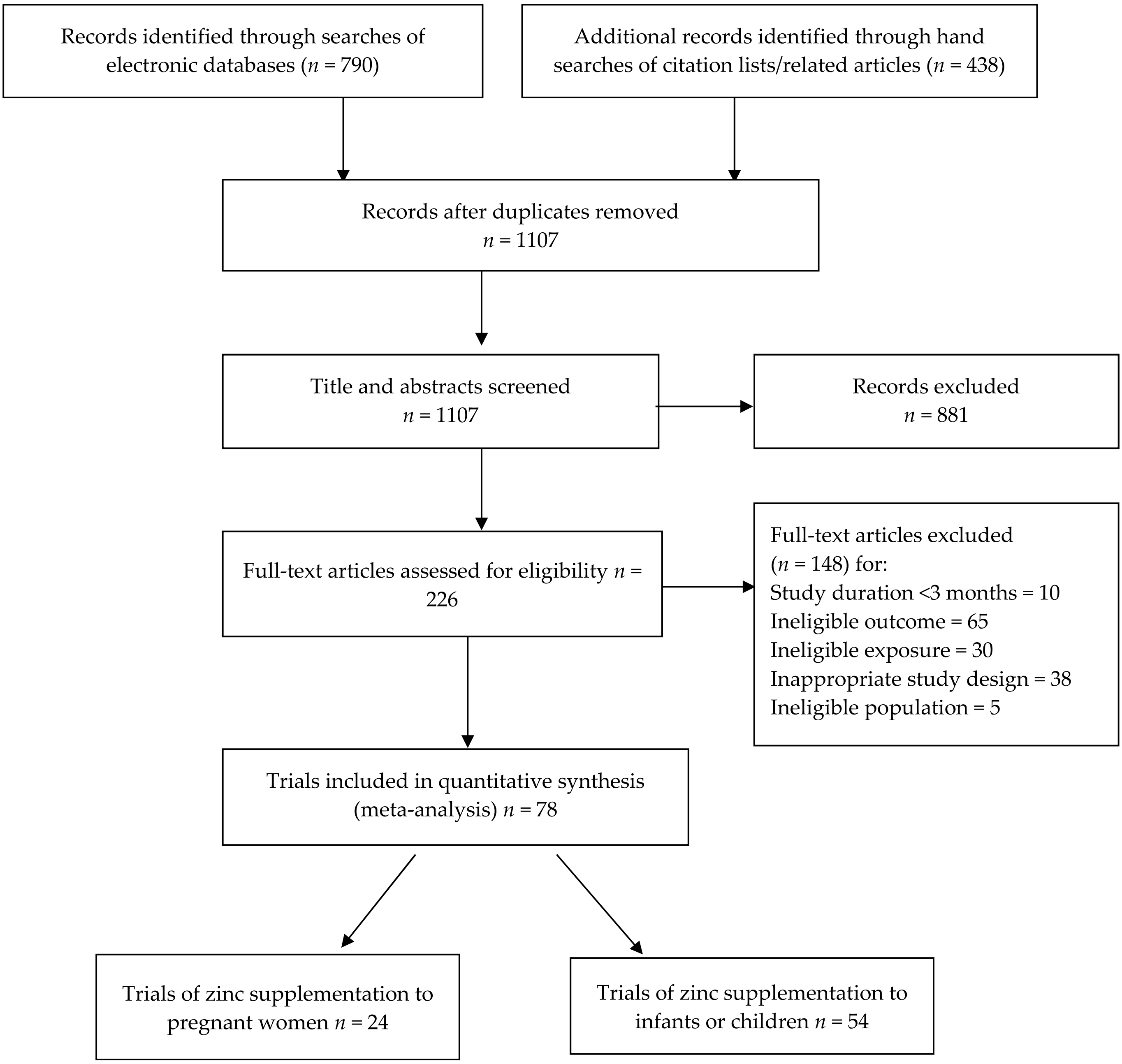

Supplementary Figure 1. PRISMA Flowchart of study selection and inclusion.

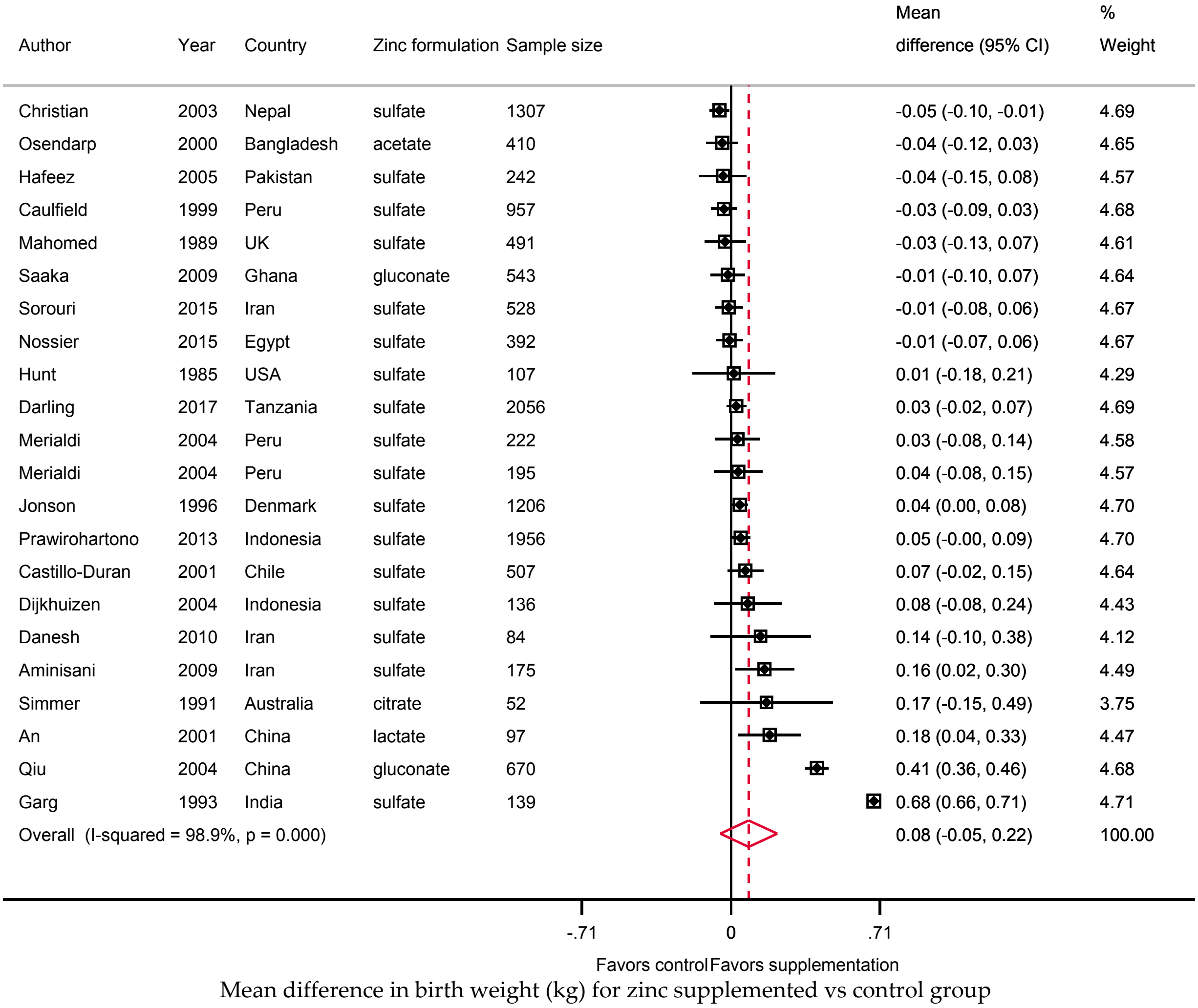

Supplementary Figure 2. Effect of zinc supplementation during pregnancy on birth weight in randomized controlled trials.

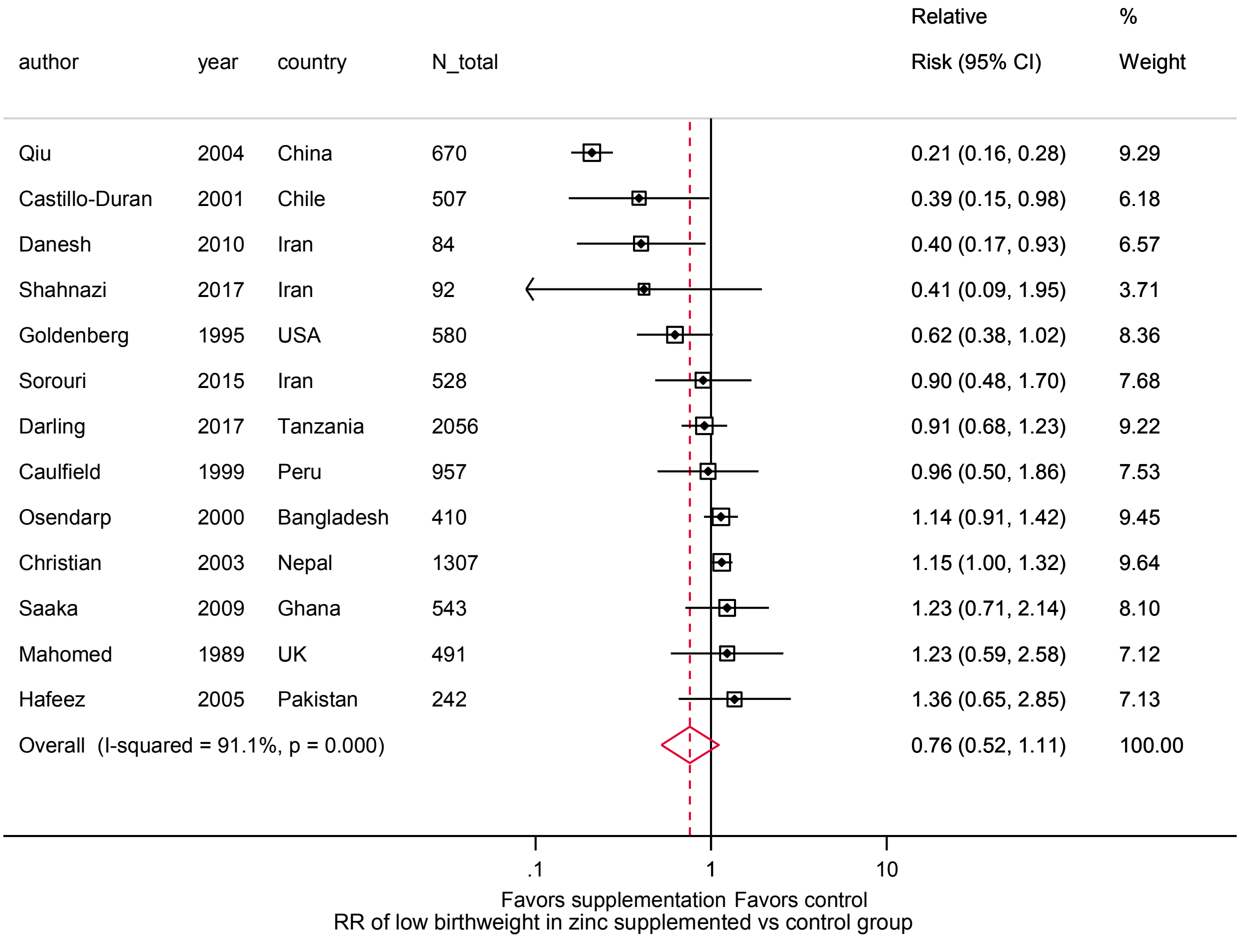

Supplementary Figure 3. Effect of zinc supplementation during pregnancy on low birth weight in randomized controlled trials.

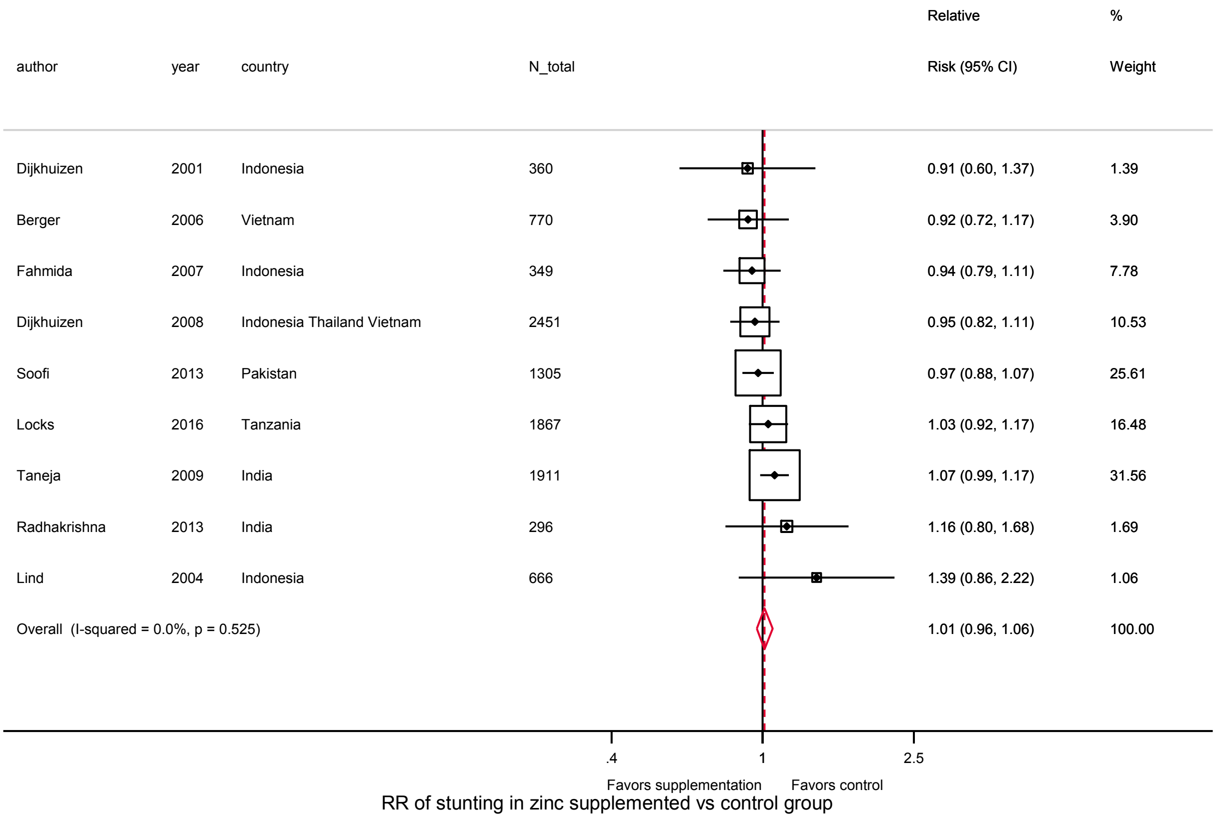

Supplementary Figure 4. Effect of zinc supplementation among children <5 y old on risk of stunting in randomized controlled trials.

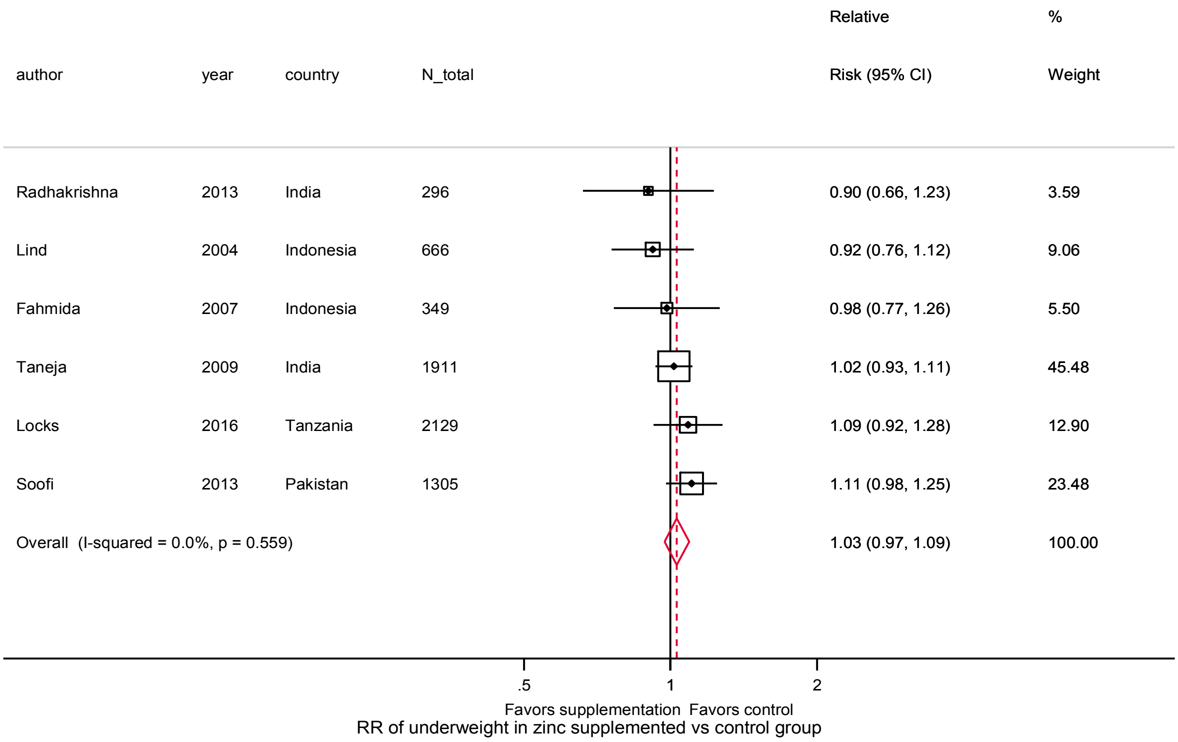

Supplementary Figure 5. Effect of zinc supplementation among children <5 y old on risk of underweight in randomized controlled trials.

.

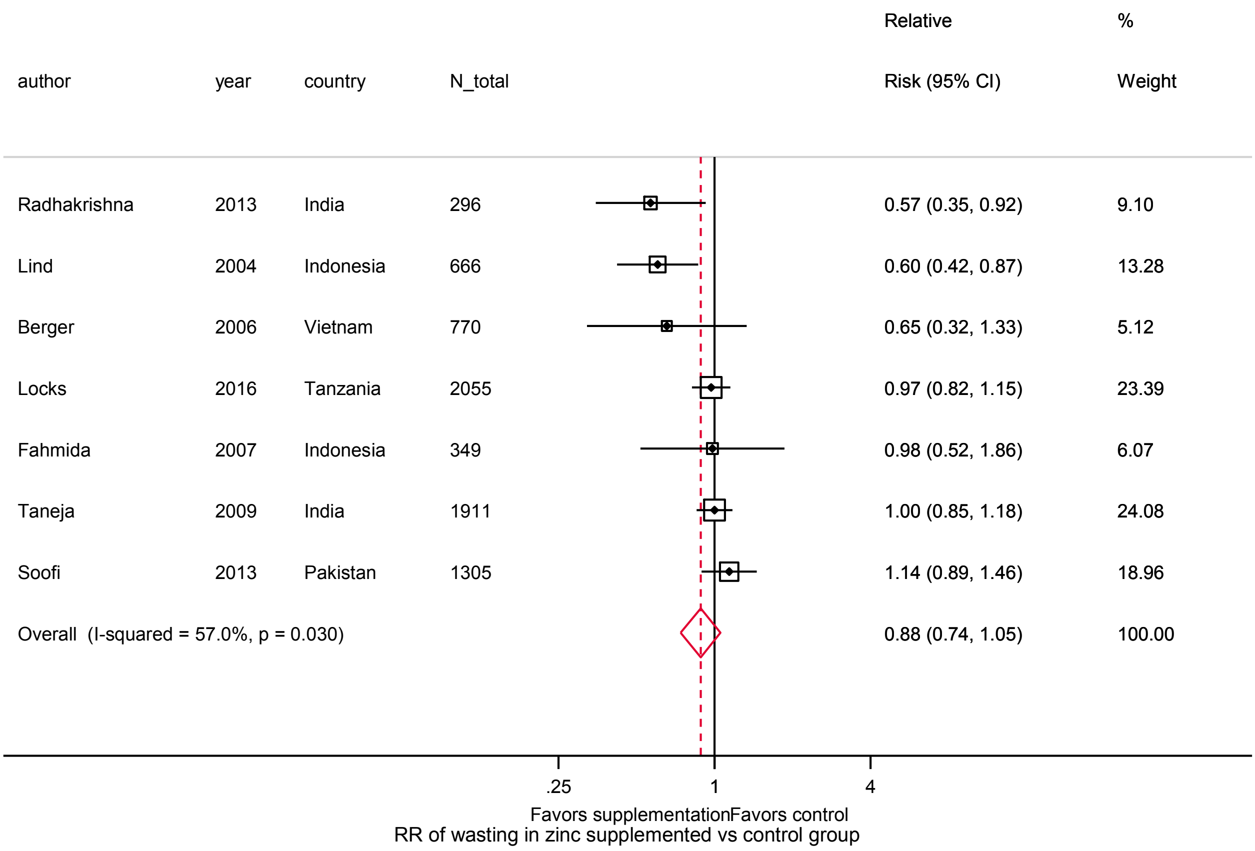

Supplementary Figure 6. Effect of zinc supplementation among children <5 y old on risk of wasting in randomized controlled trials.

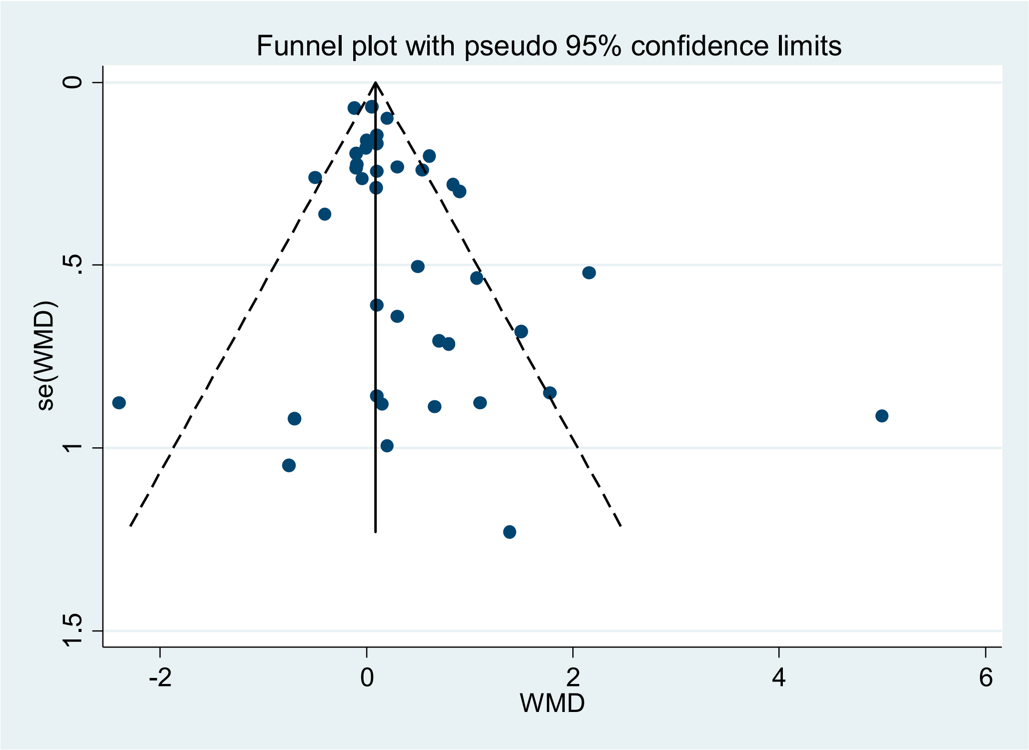

(A) Height, P=0.01

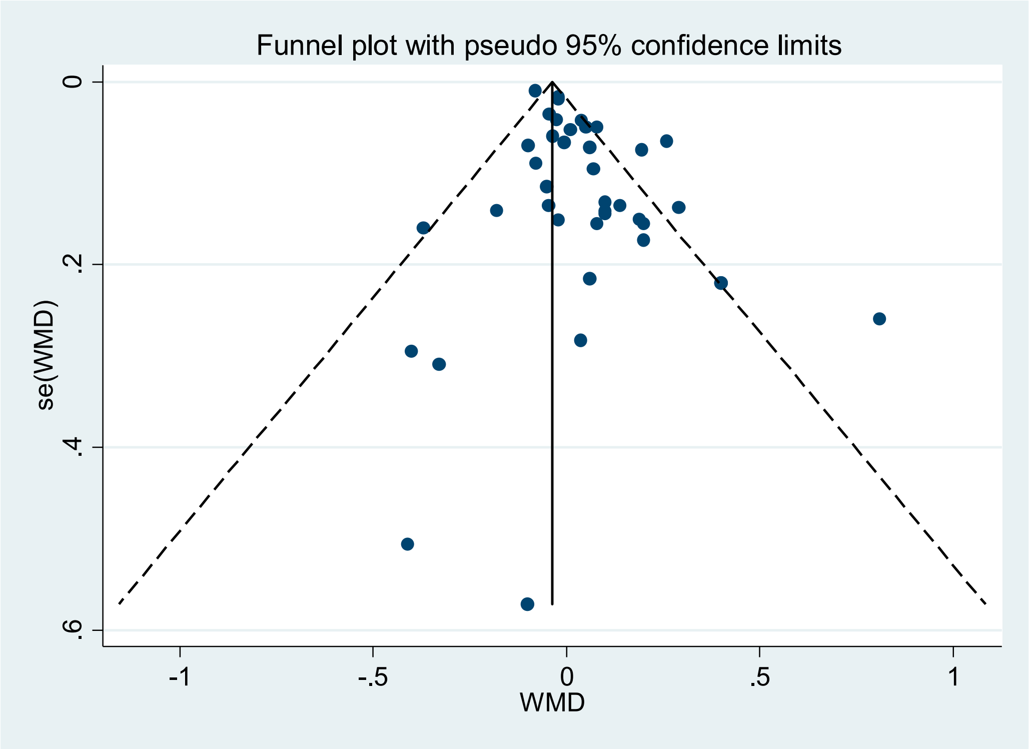

(B) HAZ, P<0.001

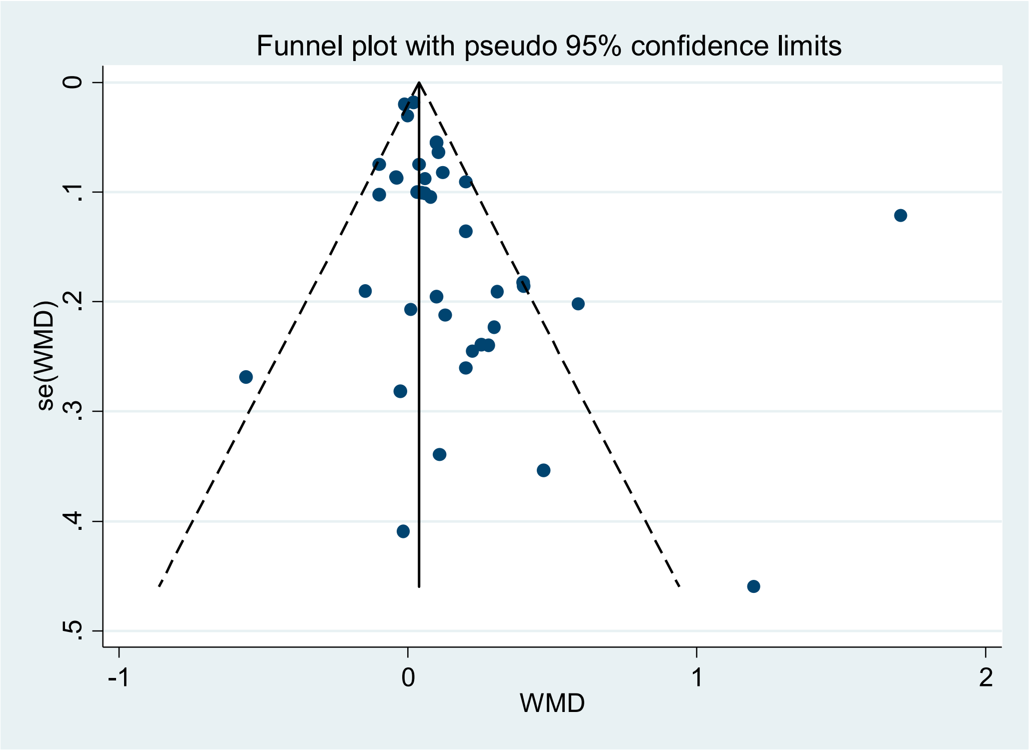

(C) Weight, P=0.03

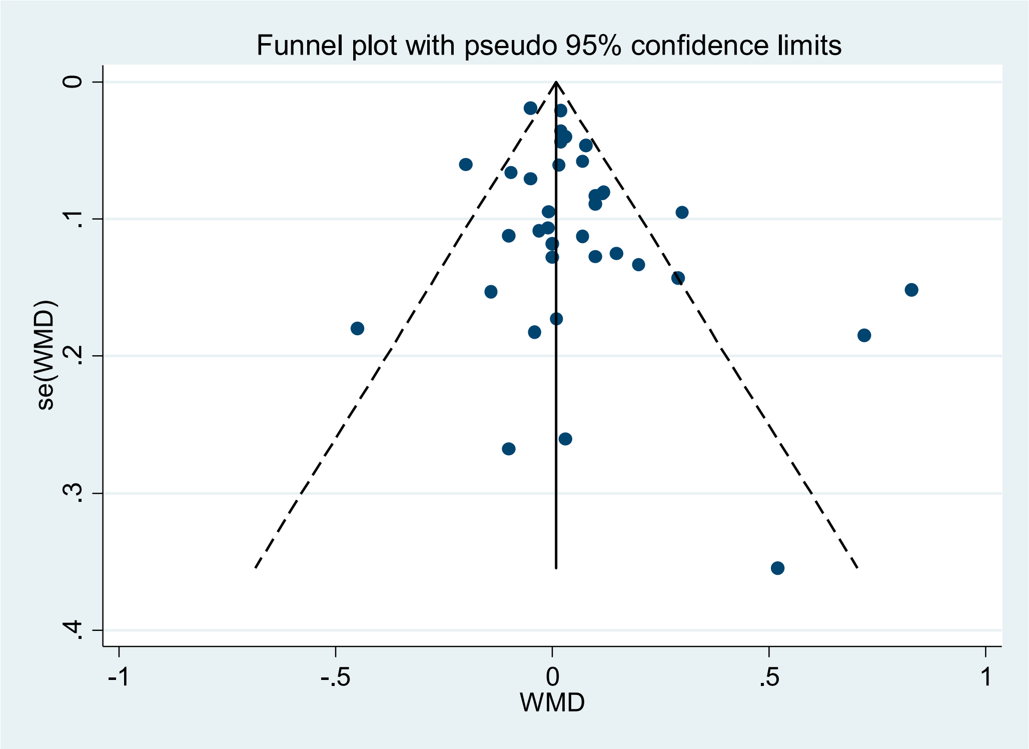

(D) WAZ, P=0.04

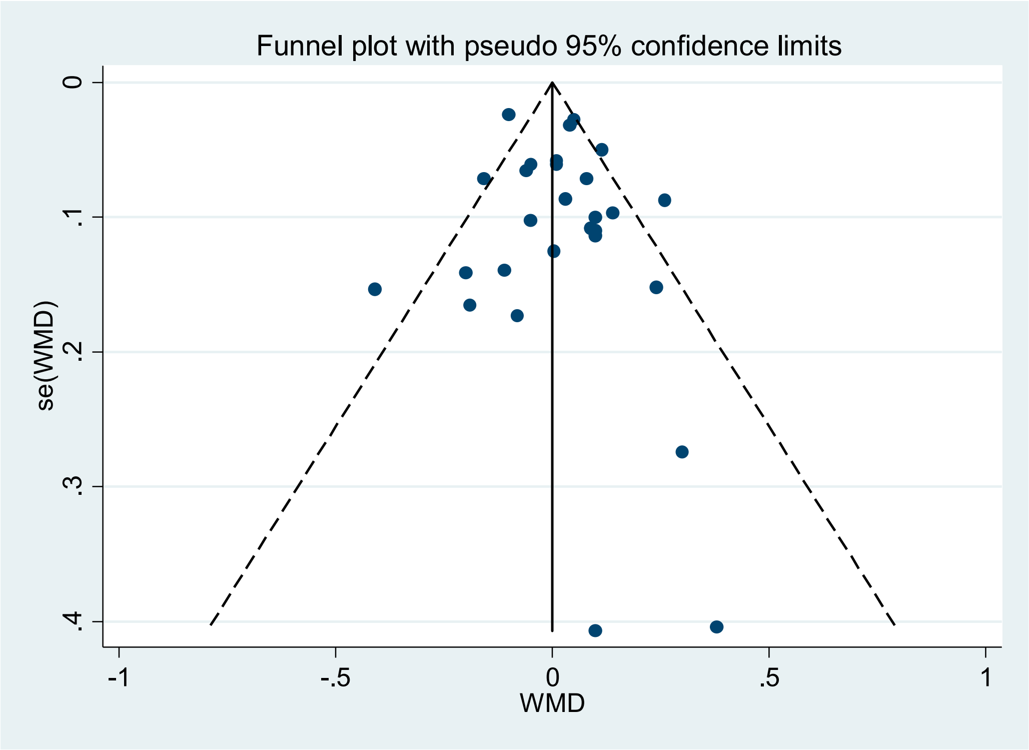

(E) WHZ, P=0.36

Supplementary Figure 7. Funnel plots for height (A), HAZ (B), weight (C),WAZ (D), and WHZ (E). P values are from Egger's test, which evaluates asymmetry of funnel plot of studies based on a linear regression of normalized effect estimate (estimated divided by its standard error) against precision (reciprocal of the standard error of the estimate).

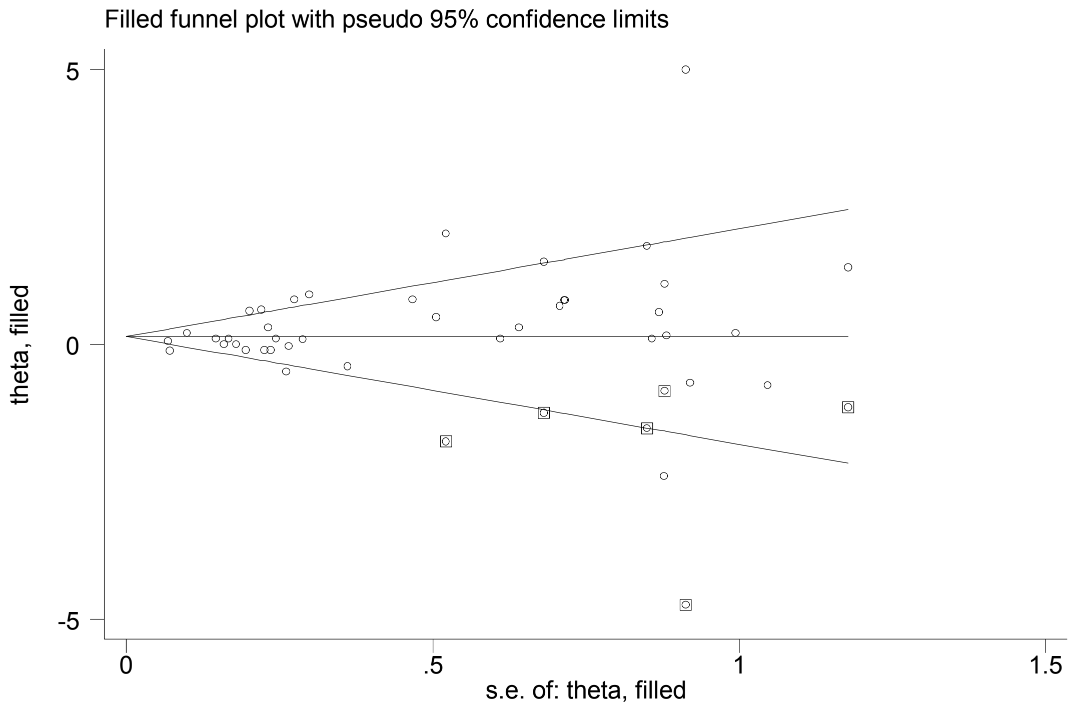

(a) Height

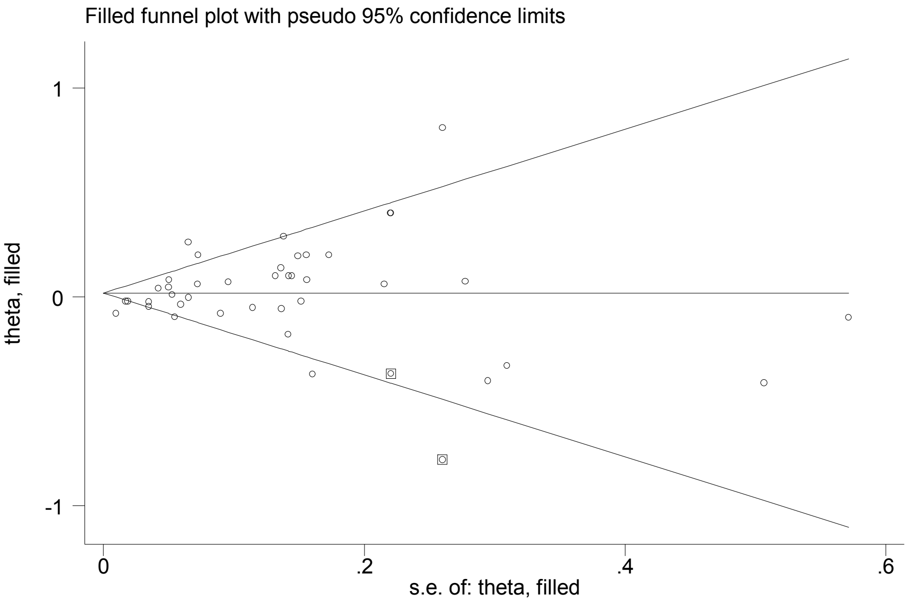

(b) HAZ

Supplementary Figure 8. Filled funnel plots for (a) height and (b) HAZ using Duval and Tweedie's non-parametric trim-and-fill method.

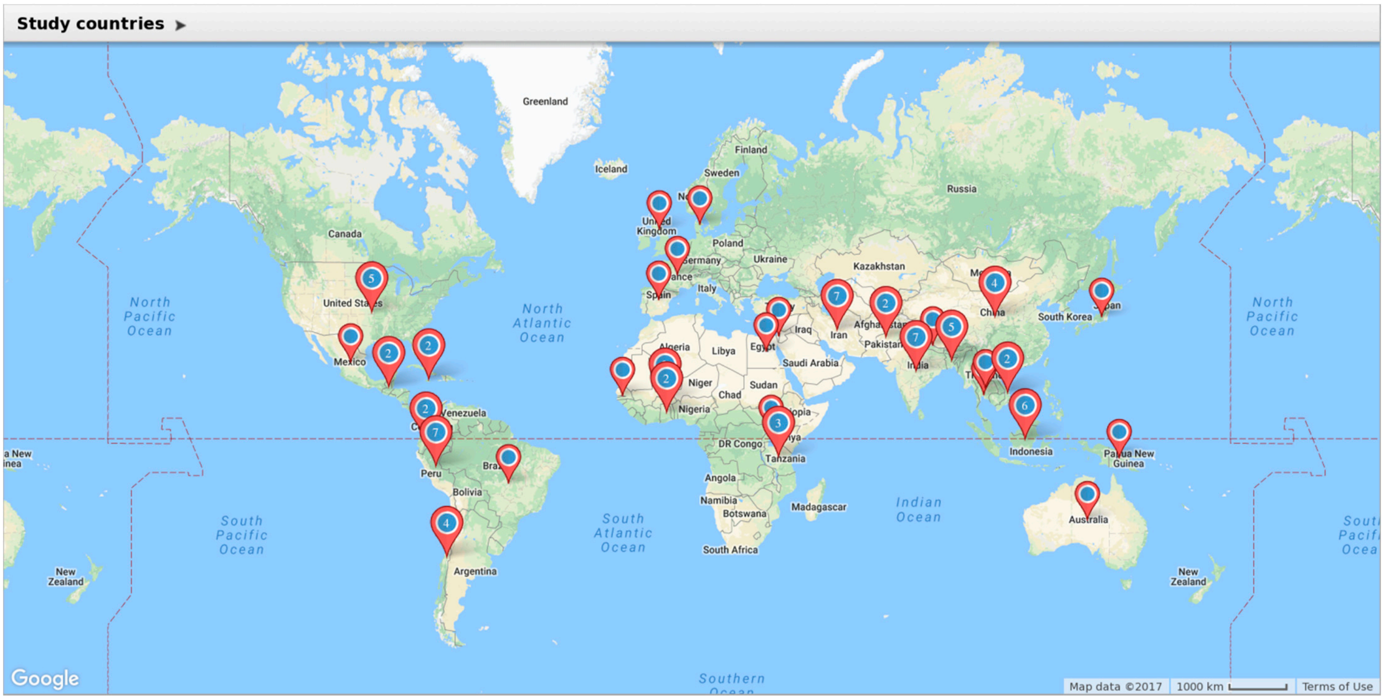

Supplementary Figure 9. A global map with all study locations (*n* = 78) included in the meta-analysis.
